# Adsorption–desorption of CO_2_ on zeolite-Y-templated carbon at various temperatures†

**DOI:** 10.1039/c8ra09200a

**Published:** 2018-12-12

**Authors:** Triyanda Gunawan, Rika Wijiyanti, Nurul Widiastuti

**Affiliations:** Department of Chemistry, Faculty of Science, Institut Teknologi Sepuluh Nopember 60111 Surabaya Indonesia nurul_widiastuti@chem.its.ac.id

## Abstract

This study aims to investigate the adsorption–desorption of CO_2_ on a micro-mesoporous zeolite-Y-templated carbon (ZTC) at various temperatures. ZTC was synthesized *via* sucrose impregnation, carbonization, and template removal. The adsorption–desorption of CO_2_ on ZTC was performed using the gravimetric method. Results showed that the CO_2_ adsorption capacity was 9.51 wt%, 5.60 wt%, and 3.47 wt%, and desorbed up to 59.83%, 69.70%, 77.5% for temperatures of 30 °C, 40 °C, and 50 °C, respectively. The adsorption process of CO_2_ at temperatures of 30 °C and 40 °C follow the pseudo-second order, while at 50 °C follows intra-particle diffusion. The thermodynamic analyses indicate that the adsorption was due to physisorption.

## Abstract 2

A micro-mesoporous structure of ZTC was synthesized *via* an impregnation method, and the structure assisted in a faster CO_2_ adsorption–desorption equilibrium.

## Graphical abstract

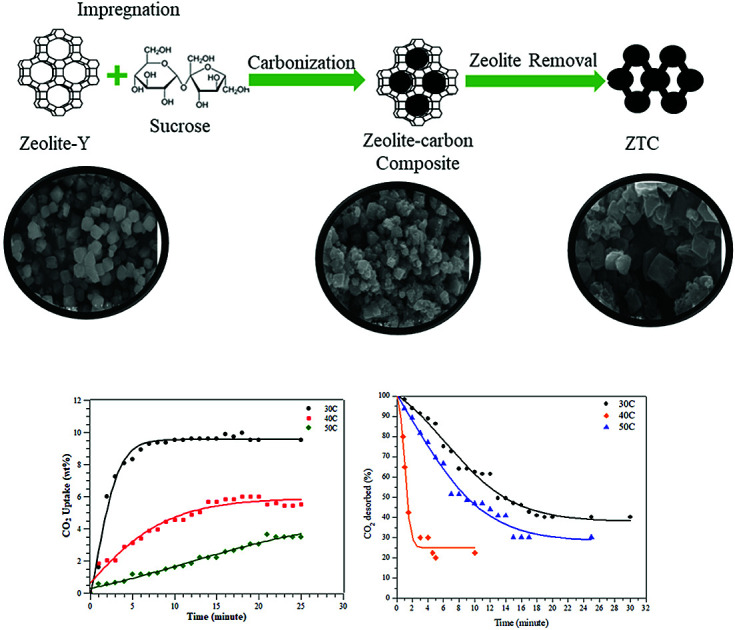

## Introduction

Global warming caused by greenhouse gas emissions has attracted some researchers to solve this issue. CO_2_ is the main contributor to global warming with a 60% contribution among all greenhouse gasses.^1–3^ This could be because this gas is emitted in large amounts by industrial processes and from the combustion of fuels.^4^ The CO_2_ concentration in our atmosphere nowadays is nearly 400 ppm, which is way higher than that of the pre-industrial era, which was 300 ppm.^5^ Moreover, the presence of CO_2_ in a natural gas source reduced the heat value. Thus, there is a strong desire to reduce the CO_2_ concentration. Carbon capture and storage (CCS) is one approach to reduce this greenhouse gas.^1–3^

In the past, CO_2_ was captured by utilizing various ethanolamine solutions *via* a chemical absorption mechanism. However, this application required high operating costs because the absorbent was lost very easily and because the application required a lot of energy.^6^ Adsorption by a porous solid is a promising alternative to this issue in terms of the energy saving and ease of operation. There are three main requirements to develop a CO_2_ adsorbent: high adsorption capacity of CO_2_, adequate adsorption/desorption kinetics for carbon dioxide at operating conditions, and long-lasting after a repeated adsorption/desorption cycle.^4,7–9^

Carbons, zeolites, ordered mesoporous materials, and silica are physisorption-based materials that are mostly used for CO_2_ capture today.^10^ Among those materials, zeolites and carbons are the most interesting. Zeolite is an alumina-silicate material, which has a high microporosity, and its pore surface is easily adjusted depending on the desired application. The pores can be micropores (<2 nm) or mesopores (2–50 nm), and are well distributed. However, a zeolite has the disadvantage that its surface area is lower than that of carbon materials and is easily poisoned by CO_2_ in the presence of moisture.^7,11^ On the other hand, carbon materials have high specific surface areas up to 300–1000 m^2^ g^−1^ or even more, while their porosities are lower than that of a zeolite. Zeolite has a cage-like pore structure with a molecular size of 0.5–1.2 nm. A high porosity zeolite can capture 0.45–6.52 mmol g^−1^ CO_2_ at room temperature, while high surface carbon can capture 21.29 mmol g^−1^ CO_2_ at room temperature.^12^ Based on these research, the idea of combining both materials arose to produce a material that has the advantages of both materials. It overcomes the shortcomings of the two materials, so that high porosity and high surface area can be obtained in one material to maximize the adsorption of CO_2_. This material can be synthesized using a carbon template zeolite method.

The pore structure formed in the as-synthesized carbon template zeolite depends on the synthetic conditions.^13,14^ One of the simplest methods for synthesizing this material is using an impregnation method.^15,16^ Basically, this method is conducted by adding a carbon precursor into the pore of a hard template material, followed by carbonization at 500–1000 °C under N_2_ flow and finally undergo an acid treatment to remove the zeolite template. In a previous study, Zhou *et al.* and Youn *et al.* utilized zeolite-Y as a hard template to produce a high microporosity ZTC.^17,18^ The materials exhibited a large amount of adsorbed CO_2_ capacity, up to 9.3 wt% at room temperature and a high pressure of 40–100 kPa. However, the literature discussing detailed CO_2_ adsorption–desorption at room pressure and temperature is limited.

In the present study, the adsorption–desorption of CO_2_ on ZTC was studied. ZTC was prepared by an impregnation carbon source inside a zeolite-Y pore. Sucrose was used as a carbon precursor due to its high availability, high carbon residue and low cost, compared to other carbon sources.^19,20^ The CO_2_ adsorption–desorption was conducted at 30 °C, 40 °C and 50 °C at a pressure of 1 bar. A thermodynamic and kinetic study was undertaken to observe the gas transport mechanisms.

## Experimental

### Materials

Sulfuric acid (H_2_SO_4_, 98%) was purchased from Merck. Sodium aluminate (NaAlO_2_, Sigma Aldrich) was used as an aluminate and sodium source for zeolite formation. Sodium silicate (Na_2_SiO_3_, Sigma Aldrich) was used as a silicate and sodium source and sodium hydroxide (99% NaOH, pellet) was purchased from Sigma Aldrich for an additional sodium source and as a counter ion of zeolite. Sucrose (98%, Fluka) was used as a carbon source for ZTC.

### Procedure

#### Synthesis of zeolite-Y

**Fig. 1:**
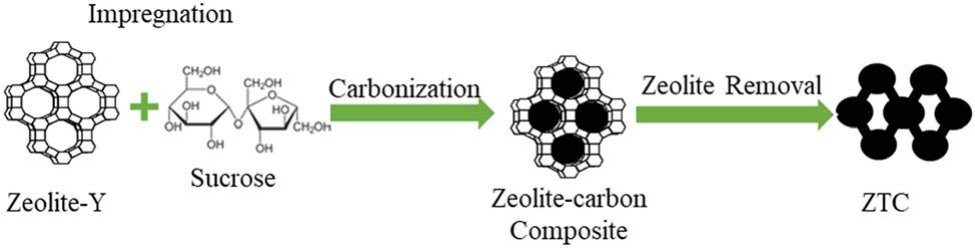
Synthesis route of ZTC.

The overall synthesis route of ZTC can be seen in Fig. 1. Zeolite-Y was synthesized *via* a hydrothermal reaction of zeolite seed crystals. The molar composition of the gel was 1.0NaAlO_2_ : 18Na_2_SiO_3_ : 1.75NaOH : 23.33H_2_O.^21^ The gel was prepared by dissolving NaOH and NaAlO_2_ into DI water. The solution was stirred at room temperature until a homogenous aluminate solution was obtained. Then, Na_2_SiO_3_ was added dropwise into the aluminate solution and stirred for 20 minutes. The solution was then moved into a stainless steel autoclave and aged for 1 day. The hydrothermal reaction was conducted in an oven at 100 °C for 7 h. The zeolite was then filtered and washed until the pH of the filters reached <9, followed by drying at 110 °C for 24 h. The white powder zeolite mass was then measured and stored in a desiccator for the next purpose.

#### Synthesis of zeolite-Y-templated carbon

The as-synthesized zeolite-Y was used as a hard template for preparing the ZTC. First, the zeolite–carbon composite was synthesized *via* an impregnation method.^19^ Zeolite-Y was degassed at 200 °C for 4 h inside a homemade tubular furnace with a heating rate of 1 °C min^−1^ to remove any adsorbed gasses prior to the impregnation process. The mass ratio of zeolite-Y and sucrose was 1 : 1.25. The impregnation process was carried out by dissolving sucrose into 50 mL of 0.35 M H_2_SO_4_. The zeolite Y has a high resistance toward sulfuric acid, up to 5 M of sulfuric acid.^22^ Then, degassed zeolite-Y was added and the solution was stirred at 250 rpm for 72 h at room temperature. The solution was then filtered and its residue (the impregnated zeolite) was moved into a tubular holder prior to the carbonization process. The pyrolysis process was conducted inside a homemade tubular furnace at 800 °C for 4 h with a heating rate of 2 °C min^−1^, under a N_2_ flow of 30 cm^3^ min^−1^. The zeolite-carbon composite was then ground to obtain a fine powder and immersed in 5% HF for 1 h to break the Si bonding in the zeolite. The Al phase was removed by refluxing the sorbent at 60 °C in 37% hydrochloric acid (HCl) and was finally immersed in 48% HF for 1 h to completely remove the zeolite template. After each acid treatment, the sample was dried at 110 °C. The ZTC obtained was then stored inside a desiccator for future treatment.

#### Sample characterization

Cu Kα radiation X-ray diffractogram (XRD) observations were performed on a Brucker D8 Advance diffractometer. The morphology of each sample was observed using a scanning electron microscope (Hitachi, TM 3000) with a potential of 15 kV and samples were coated with platinum. A high resolution transmission electron microscope (H9500) was employed to observe the carbon microstructure.

The pore properties were measured using a surface area and porosity analyzer (Micromeritics, ASAP 2020). The gas used for the adsorptive analysis was N_2_ and the analysis temperature was −195 °C. Prior to the measurement process, each sample was degassed at 300 °C overnight. The BET (Brunauer–Emmett–Teller) method was utilized to calculate the surface area (*S*_BET_) in the relative pressure range of 0.05 to 0.25. The total pore volume (*V*_T_) was obtained at *P*/*P*_0_ = 0.995. The *t*-plot method was utilized to calculate the micropore volume (*V*_micro_), while subtracting the total pore volume from the result obtained from the *t*-plot will give the external volume (*V*_ext_). The pore size distribution (PSD) was obtained using SAIEUS software with 2D-NLDFT.^23^

The adsorption–desorption of CO_2_ was examined using a gravimetric method. 1 g of the ZTC sample was dried at 105 °C for 2 h. The ZTC was then cooled at room temperature and stored inside a desiccator. The adsorption–desorption process was conducted at temperatures of 30 °C, 40 °C, and 50 °C.

### CO_2_ adsorption–desorption measurement

#### Gravimetric measurement

**Fig. 2:**
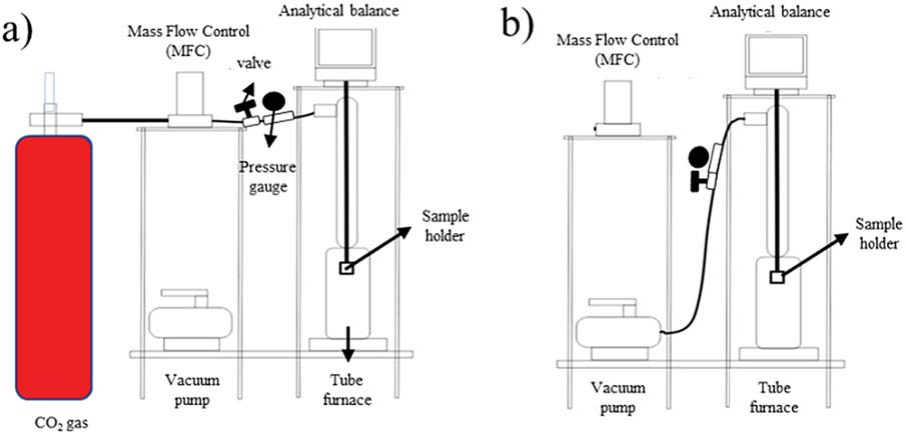
Schematic diagram of the (a) adsorption system and (b) desorption system.

The gravimetric measurement was conducted on an in-house gravimetric apparatus. 1.3 g of the sample was degassed at 350 °C for 3 h prior to the adsorption–desorption measurement. The adsorption–desorption process was conducted at temperatures of 30 °C, 40 °C, and 50 °C under a pressure of 1 bar using three different fresh samples. The CO_2_ flow rate during the adsorption process was controlled at 20 mL min^−1^. The desorption measurements were conducted simultaneously after the adsorption reached equilibrium and were assisted by vacuum. The alteration of mass was recorded by the Ohaus Pioneer analytical balance. The adsorption–desorption system used in this study is illustrated in Fig. 2.

Eqn (1) was used to determine the weight of adsorbed CO_2_:1
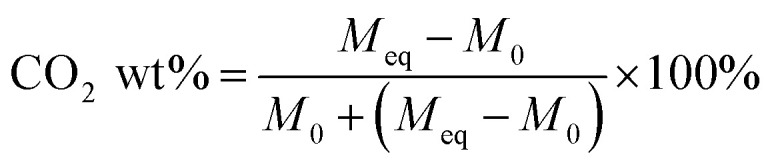
where *M*_eq_ is the mass after adsorption reached equilibrium and *M*_0_ is the initial mass of the adsorbent after the degassing process. Eqn (2) was utilized for the desorption process:2
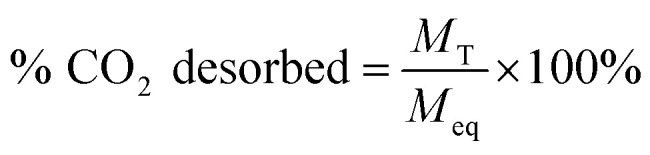
where *M*_T_ is the remaining mass of *M*_eq_ at time *T*. Each measurement was repeated five times to ensure the accuracy of the data.

### Adsorption kinetics

Kinetic models are used to determine gas transport mechanisms and adsorption types of CO_2_ into ZTC. In this study, pseudo-first order, pseudo-second order, and intra-particle diffusion models were used.^24,25^

#### Pseudo-first order

Generally, the model used to describe the adsorption process is given by eqn (3):

3 ln(*q*_e_ − *q*_*t*_) = ln *q*_e_ − *k*_1_*t*

where *q*_*t*_ (mmol g^−1^) is the amount of adsorbate adsorbed at the time of *t* (minute), *q*_e_ (mmol g^−1^) is the adsorption capacity at equilibrium and *k*_1_ (min^−1^) is the rate constant of the pseudo-first order.

#### Pseudo-second order

The pseudo-second order can be determined using eqn (4):4
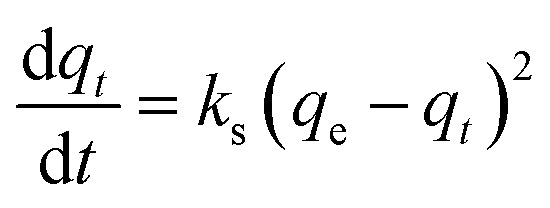


After integrating and applying boundary conditions, the equation can be integrated further with the initial condition of *q*_*t*_ = 0 at *t* = 0 and *q*_*t*_ = *q*_*t*_ at *t* = *t*. A linear equation can be obtained5
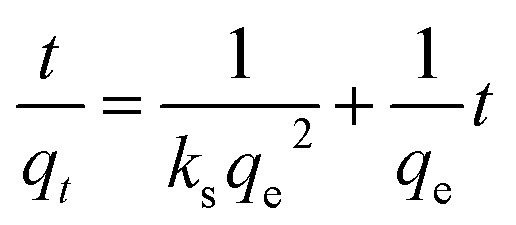
and the initial sorption rate, *h* (mg g^−1^ min^−1^) as *t* → 0 can be defined as

6 *h* = *k*_s_*q*_e_^2^

#### Intra-particle diffusion

Intra-particle diffusion was used to determine the adsorption process in porous materials and is expressed in eqn (7):7
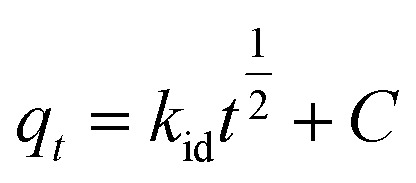
where, *t* is time (min). The diffusion constant *k*_id_ (mmol g^−1^ min^−0.5^) can be determined experimentally from the slope of the plot of *q*_*t*_*versus t*^1/2^, and *C* is an intercept that expresses the thickness of the boundary layer.^1–3^

### Thermodynamics adsorption

A thermodynamic study was conducted to confirm the kinetic study and to determine the adsorption characteristics of CO_2_ into ZTC. The thermodynamic parameters were determined using the van't Hoff equation, given in eqn (8).8
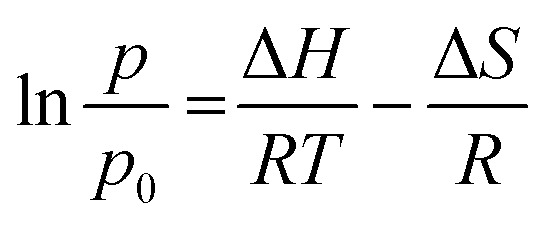
where Δ*H* is enthalpy adsorption (kJ mol^−1^), *T* is temperature (K), *p* is pressure at the equilibrium state (bar), *q* is the adsorption capacity at the equilibrium state, and *R* is a gas constant (8.314 J mol^−1^ K^−1^).

## Result and discussion

### Synthesis of zeolite-Y-templated carbon

**Fig. 3:**
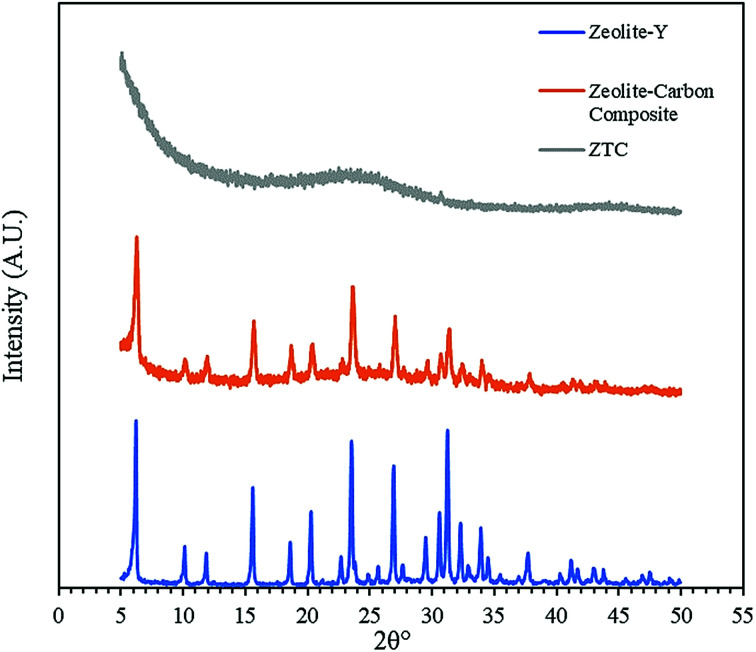
Diffractogram patterns of the as-prepared samples.

Fig. 3 shows the diffractogram pattern of the prepared carbon sample and the corresponding zeolite template used in this study. The (111) planes of the zeolite-Y crystal were observed in a high peak around ∼6°, indicating a highly arranged zeolite crystal formation. The peak remains intact even after impregnation in acid and pyrolysis, since zeolite-Ys have high resistance toward sulfuric acid up to 5 M,^22^ indicating that the structure of the zeolite-Y did not change during the process. However, the intensity was reduced after the carbon filling processes, but the intensity of the other peaks did not change significantly. On the other hand, the peak at ∼6° was not found in the diffractogram pattern of the ZTC. This indicates low replication of the carbon to the template structure. This result was also observed previously.^19^ A broad, weak peak in the 2*θ* range of 20–25° was observed. This is a reflection of (002) mesophase graphite-like material and often appears in such material.^17,18^ Interestingly, wide angle peaks at ∼43°, which correspond to the (101) of graphitic carbon, were not observed in this sample. This indicates that the ZTC material still replicated the structure of a zeolite body and even possesses mesoporous characteristics.

**Fig. 4:**
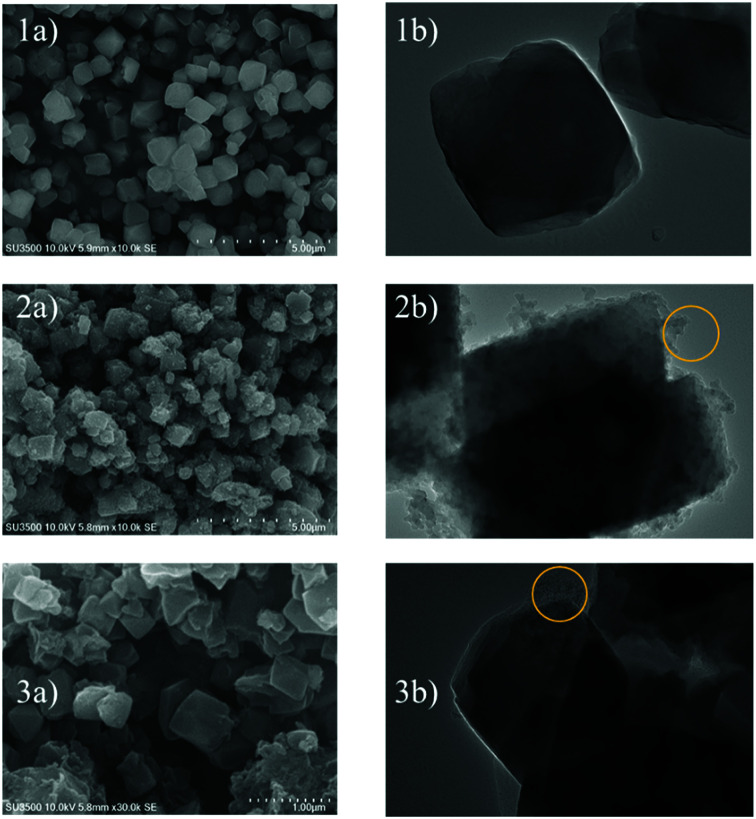
SEM (a) and TEM (b) images of zeolite-Y (1), composite carbon (2), and ZTC (3). The yellow circles correspond to the external graphitic carbon.

**Fig. 5:**
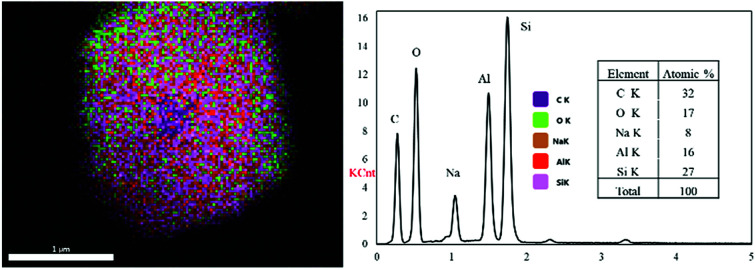
The EDX mapping image of the composite (left) and the atomic distribution in it (right).

Fig. 4 shows the SEM and TEM images of the prepared samples. As shown in Fig. 4(1a), the morphology of zeolite-Y exhibits a typical crystal-like structure with an octahedral configuration, and the particle size was about 400–600 nm. The same morphology was also observed for the composite and ZTC sample. However, a thin layer of graphitic carbon was observed on the surface of both the composite and ZTC, as marked in yellow circles on their respective TEM images. We suggest this layer is the reason for the broad, weak peak in the 2*θ* range of 20–25° that appeared in the diffractogram data of ZTC. Moreover, to observe the element distribution after the impregnation and pyrolysis process, a TEM + EDX observation was conducted, as shown in Fig. 5. As can be seen in the EDX mapping, the carbon distribution was mainly inside the zeolite body, with a small amount of it on the outer side of the zeolite. This result confirms the XRD and SEM results discussed previously.

**Fig. 6:**
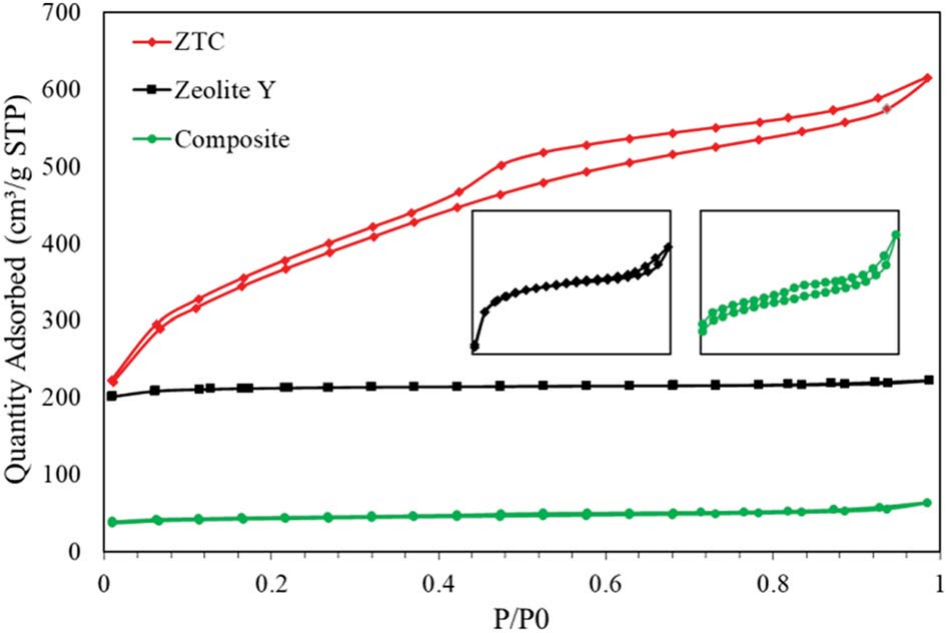
The N_2_ isotherm graph of zeolite-Y, composite and ZTC.

Fig. 6 shows the N_2_ isotherms of all the as-prepared samples. All the samples show typical type 1 adsorption, which indicates microporous materials. However, in both the composite and ZTC, type H4 hysteresis was observed. This hysteresis suggests the presence of mesopores in both material and narrow slit pores.^26^ The specific surface area (*S*_BET_) of the ZTC was the highest of them all, reaching up to 1254.38 m^2^ g^−1^, almost double the *S*_BET_ of zeolite-Y, which was 678.48 m^2^ g^−1^. In contrast, the *S*_BET_ of the composite was the smallest of them all, which was only 133.29 m^2^ g^−1^. The reduction of the *S*_BET_ in the composite was because the pore of the zeolite was filled by carbon from the sucrose precursor. This indicates that the impregnation process was successful. The *S*_BET_ trend was followed by the pore volume result. The pore volumes of the zeolite-Y, composite, and ZTC were 0.344 cm^3^ g^−1^, 0.098 cm^3^ g^−1^, and 0.945 cm^3^ g^−1^, respectively. These results proved that carbon preparation through a simple method of impregnation using a zeolite as a hard template will produce carbon that has a high pore volume and surface area.

**Fig. 7:**
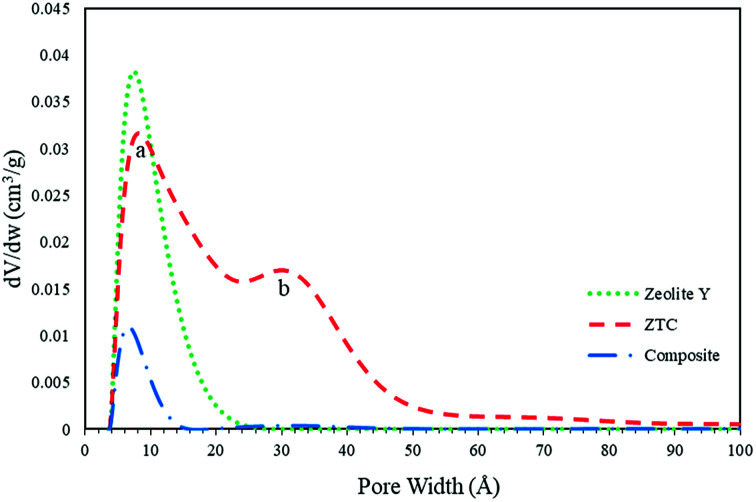
The PSD obtained from 2D-NLDFT calculations.

The pore size distribution (PSD) was determined using SAIEUS software with 2D-NLDFT. The result is illustrated in Fig. 6. As can be seen in Fig. 7, the PSD of both the zeolite-Y and composite show a sharp peak, indicating narrow pore size distributions. The only big difference between these two graphs was the peak intensity (volume). The peak intensity of the zeolite is higher. The presence of carbon inside the zeolite pore not only reduced the pore volume significantly, but also reduced the average pore size of the zeolite-Y from 8.61 ± 0.07 Å to 7.29 ± 0.04 Å. The small pore of the composite has potential in applications that do not rely much on surface area and pore volume, such as for separation processes. On the other hand, the PSD of ZTC showed abroad peak, which was divided into two regions, a and b. Region a was microporous region with an average pore diameter of 9.23 ± 0.10 Å, and the size of this region was almost twice that of region b. Region b corresponds to the mesoporous site with an average pore diameter of 24.55 ± 0.84 Å. Moreover, the overall average pore size of ZTC was in the mesopore region which originated from imperfect sucrose impregnation. It can be concluded that the mesoporous region lies in the outer part of ZTC. This result was in agreement with the XRD, SEM, and TEM results discussed above. The presence of high micropores in ZTC would improve the CO_2_ adsorption, while the presence of mesopores would speed up the adsorption process.^27^ The physical characteristics of all samples are summarized in Table 1.

**Table:** The physical properties of all samples

Parameters	ZTC	Composite	Zeolite-Y
*S*_BET_ (m^2^ g^−1^)	1254.38	133.29	678.48
*t*-Plot micropore area	1051.72	96	620
Pore volume (cm^3^ g^−1^)	0.95	0.10	0.34
Average pore size (nm)	1.55 ± 0.64	0.73 ± 0.04	0.86 ± 0.07

### CO_2_ adsorption

**Fig. 8:**
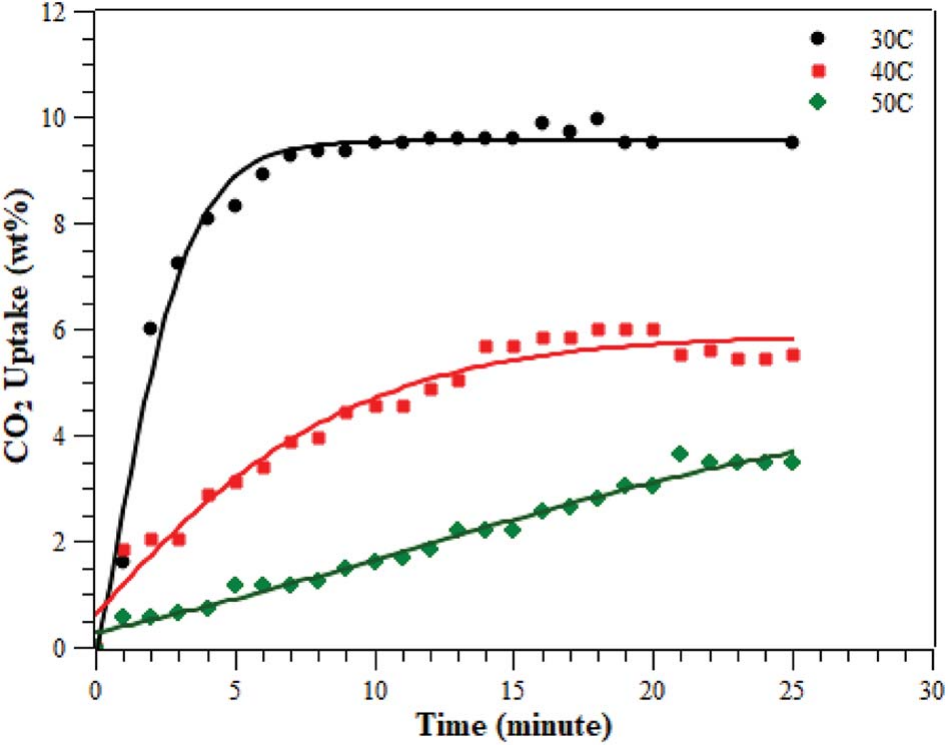
The adsorption of CO_2_ on ZTC at various temperatures under a pressure of 1 bar.

The CO_2_ adsorption was conducted at various temperatures (30 °C, 40 °C, and 50 °C) and 1 bar. The adsorption measurement results can be seen in Fig. 8 and Table 2. The results showed that the adsorption conducted at 30 °C had the greatest adsorption CO_2_ capacity of 9.51 ± 0.48 wt%, followed by the adsorption at 40 °C and then at 50 °C of 5.60 ± 0.28 and 3.47 ± 0.17 wt%, respectively. This indicates that CO_2_ adsorption occurs preferentially at a lower temperature.^3,28–30^ The CO_2_ adsorption capacity at 30 °C was higher than at the other two temperatures, due to physisorption and the fact that CO_2_ adsorption on porous materials requires a matching of pore sizes. Since the PSD of the ZTC mainly consisted of micropores, it had a high CO_2_ uptake due to pore matching with the CO_2_ molecular size. It was already confirmed that CO_2_ uptake is only determined by pores smaller than a certain diameter, not by the total pore volume.^3,27^ Moreover, the temperature-dependent size also plays an important role for CO_2_ sorption. Zhang *et al.* reported that the CO_2_ adsorption capacity at 75 °C, 25 °C and 0 °C and a pressure of 1 bar was determined by micropores of 0.54 nm, 0.7 nm, and 0.8 nm in size, respectively.^31^ Since the ZTC studied here possesses mesopores that make up 50% of the amount of micropores, the adsorption at elevated temperatures decreased significantly since there was no suitable space to which the CO_2_ molecules could attach. In summary, the adsorption at 30 °C was mainly in the micropore sites of zone (a) in the PSD curve, while the adsorption at 50 °C was mainly in the mesopore sites of zone (b). Additionally, since the pore structure of the ZTC studied here has a micro-mesoporous pore structure, it has its own benefit to enhance the transfer of CO_2_ molecules into and out of the inner microporous network.^27^

**Table:** CO_2_ adsorption on ZTC at various temperatures and a pressure of 1 bar, compared with other carbon materials

Sample	Carbon precursor	Temperature (°C) at 1 bar	Surface area (m^2^ g^−1^)	CO_2_ uptake (mmol g^−1^)	Ref.
RN-450-3	Phenolic resin (with N-doped)	0	1432	6.68	3
		25		4.64	
N0.8A80F50	Chestnut tanin	0	561^a^	3.44	28
		25		2.27	
L-600	Tree leaves	0	1146	5.86	30
		25		3.74	
NDAB3-500	Arundo donax and chitosan	0	1863	3.6	29
		25		2.1	
AC-700-0.5	Polyaniline (PANI)	0	826	6.85	31
		25		4.10	
ZTC	Sucrose	30	1254	2.39 (9.51 ± 0.48)^b^	This work
		40		1.35 (5.60 ± 0.28)^b^	
		50		0.82 (3.47 ± 0.17)^b^	

a Micropore surface area.

b Number in the bracket is for wt% unit.

**Fig. 9:**
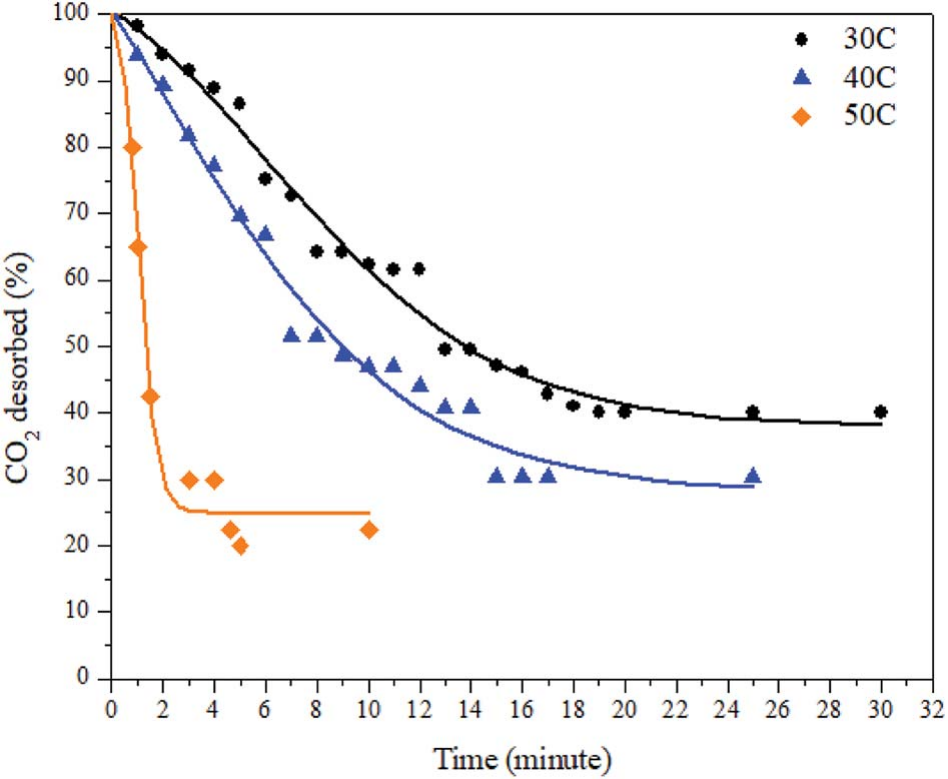
Desorption of CO_2_ on the ZTC at various temperatures and a pressure of 1 bar.

The desorption process was conducted by a gravimetric method and by using various desorption temperatures. The desorption process took place immediately after the adsorption process was completed. The decreases in mass inside the adsorbent were plotted against time. The desorption results at various temperatures can be seen in Fig. 9 and Table 3 below.

**Table:** Desorption of CO_2_ on the ZTC at various temperatures and a pressure of 1 bar^a^

Temperature (°C)	*t*_50_ (minute)	*Q*_des_ (%)
30	15	59.83
40	7	69.70
50	3	77.50

a *t*_50_, time needed to release 50% of the CO_2_. *Q*_des_, maximum amount of CO_2_ desorbed.

As can be seen in Fig. 9, the desorption capacity of CO_2_ increases as the temperature increases. As shown in Table 2, the amount of CO_2_ desorbed at 30 °C, 40 °C, and 50 °C was 59.83%, 69.70%, and 77.5%, respectively. The release of CO_2_ was slower by the time being, except at a temperature of 50 °C. The time needed to achieve desorption equilibrium is shorter at elevated temperatures. As discussed in a previous section, the desorption process can be described as the reverse process of the adsorption process. This also means that all the parameters that affect the adsorption capacity also have an effect on the desorption process. Since the adsorption at 30 °C mostly took place in the micropore sites, the desorption from this this region was the slowest because the gas movement was limited to small channels. This resulted in slower, multilayer breaking inside the pores. In contrast, the desorption at 50 °C took place faster because a small amount of CO_2_ adsorbed at the matching pore size and mostly occupied the mesopore region of the ZTC.

### Regenerative ability of the sorbent

**Fig. 10:**
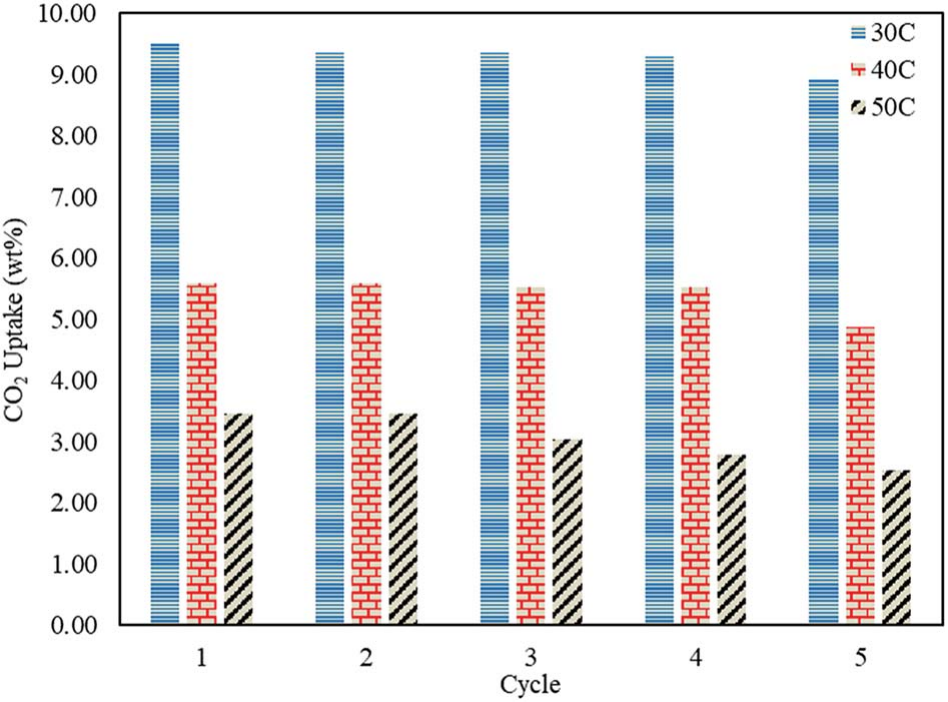
The CO_2_ uptake changes after five adsorption–desorption cycles.

**Fig. 11:**
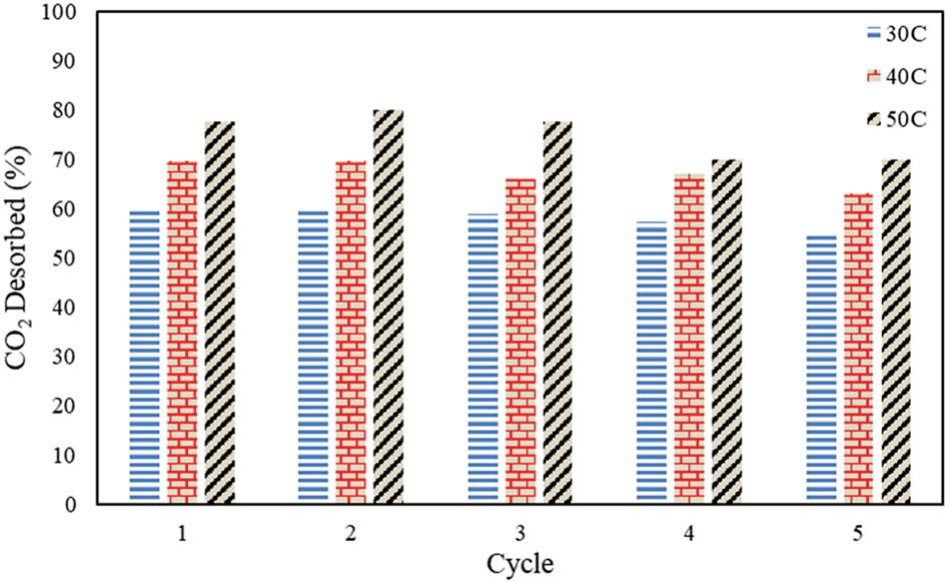
CO_2_ desorption capacity on the ZTC after five consecutive runs.

In order to study the regenerative ability of the ZTC sorbent, five simultaneous runs of the adsorption–desorption process at 30 °C, 40 °C, and 50 °C were conducted. The results are shown in Fig. 10. Prior to measurement, the initial mass of the absorbent was neglected and set to zero on an analytical balance. This allowed for easier data records of the changes in mass captured by the instrument, considered as the mass of CO_2_. All the measurements were taken at minute 30. Even though the sorbent was unable to release all of the CO_2_, as discussed in a previous section, this did not greatly affect the CO_2_ uptake capacity after five runs of the adsorption–desorption process. The notable changes between the first and fifth runs in the reduction adsorption capacity for the process conducted at 30 °C, 40 °C, and 50 °C were 6.23%, 12.97%, and 26.80%, respectively, suggesting the high potential of repeated CO_2_ capture usage at low temperature. A similar trend was also observed in the desorption process, which is shown in Fig. 11. The notable reduction in the amount of CO_2_ desorbed was 8.57%, 9.52%, and 9.68% for the process conducted at 30 °C, 40 °C, and 50 °C, respectively. The results show the great regenerative ability of the studied sorbent.

### Adsorption kinetics

**Fig. 12:**
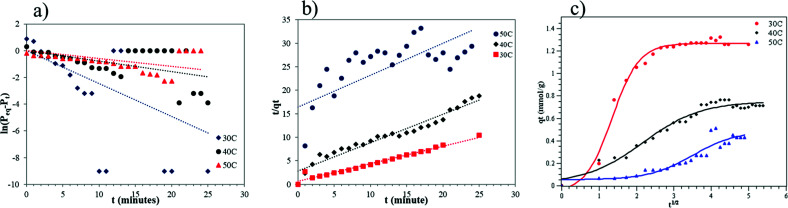
Graph of (a) pseudo-first order, (b) pseudo-second order, and (c) intra-particle diffusion models.

The results of the kinetics study can be seen in Fig. 12. The adsorption kinetics of CO_2_ gas are really important to explain the adsorption mechanism. The kinetics model used is based on the observed samples. A lot of research has reported on the adsorption kinetics of gases at solid surfaces. Most of them used pseudo-first order, pseudo-second order and intra-particle diffusion models.^3,28,29^ Therefore, in this paper, those models were used to explain the CO_2_ adsorption mechanism in the carbon template zeolite based on its physical and chemical properties, to match the experimental data, and also to study the mass transport process.

The assumption used in the pseudo-first order model is that the concentration of one reactant is much higher than the concentration of the other reactants.^25^ The pseudo-first order model's equation can be seen in eqn (1). The plot of this model is shown in Fig. 12a.

The assumption used in the pseudo-second order model is the availability of active sites on the adsorbent surface is always proportional to the adsorption capacity.^32^ The pseudo-second order's equation can be seen in eqn (3). The plot of this model is shown in Fig. 12b.

The intra-particle diffusion equation can be seen in eqn (4). By plotting *q*_*t*_ (the capacity of adsorbed CO_2_ at time, *t*, in mmol g^−1^) against *t*^1/2^, the intra-particle diffusion graph (Fig. 12c) was obtained. Fig. 12c shows two adsorption steps at 50 °C, the first one is external surface adsorption, or macropore diffusion, and the last is internal surface adsorption, or micropore diffusion. External diffusion occurs faster than internal diffusion.^33^

The important kinetic parameters are summarized in Table 3. Table 3 shows that the CO_2_ adsorption taken at temperatures of 30 °C and 40 °C follows the pseudo-second order as it has the biggest *R*^2^ value. This means that at these temperatures (30 °C and 40 °C) the adsorption capacity is a proportional to the amount of active sites/micropores available on the adsorbent surface.^29^ It showed that at these temperatures there was a lot of available active sites for CO_2_ gases. Moreover, this result also suggests that CO_2_ adsorption at the corresponding temperature was mainly controlled by physical adsorption. On the other hand, the adsorption conducted at 50 °C follows the intra-particle diffusion model as it gives the biggest *R*^2^ value. At 30 °C it can be assumed that the CO_2_ gas entered the adsorbent pores *via* two steps, the first is fast external diffusion through mesopores, followed by slow internal diffusion into the micropores of the ZTC. The experiment conducted at 50 °C did not show the same adsorption behaviour. In that case, the CO_2_ penetrated the ZTC pores with ease. The adsorption at 40 °C showed an in between behaviour, indicating that this was the point where the surface properties of the ZTC began to change due to the increase in temperature. Overall, the intra-particle diffusion model suggests that the CO_2_ mass transport into the ZTC was highly affected by the adsorption temperature. However, further investigation, such as molecular modelling is needed to study the phenomena.

In Table 4*k*_f_ is the pseudo-first order rate constant (min^−1^), *q*_e_ is the adsorption capacity at equilibrium (mmol g^−1^), *h* is the pseudo-second initial rate constant (mmol g^−1^), *k*_d_ is the diffusion rate constant (mmol g^−1^ min^−0.5^), and C is the intercept that expresses the layer boundary thickness.

Parameters of each kinetic model

**Table:** 

Model	Parameters		
Pseudo-first order	*k*_f_	*q*_e_	*R* ^2^
30 °C	−0.04	1	0.28
40 °C	−0.07	1	0.15
50 °C	−0.08	1	0.76

**Table:** 

Model	Parameters		
Pseudo-second order	*h*	*q*_e_	*R* ^2^
30 °C	1.66	2.66	0.97
40 °C	0.36	1.65	0.95
50 °C	0.06	1.47	0.47

**Table:** 

Model	Parameters		
Intra-particle diffusion	*k*_d_	*C*	*R* ^2^
30 °C	0.24	0.38	0.71
40 °C	0.13	0.10	0.88
50 °C	0.12	−0.17	0.92

### Thermodynamic adsorption

**Fig. 13:**
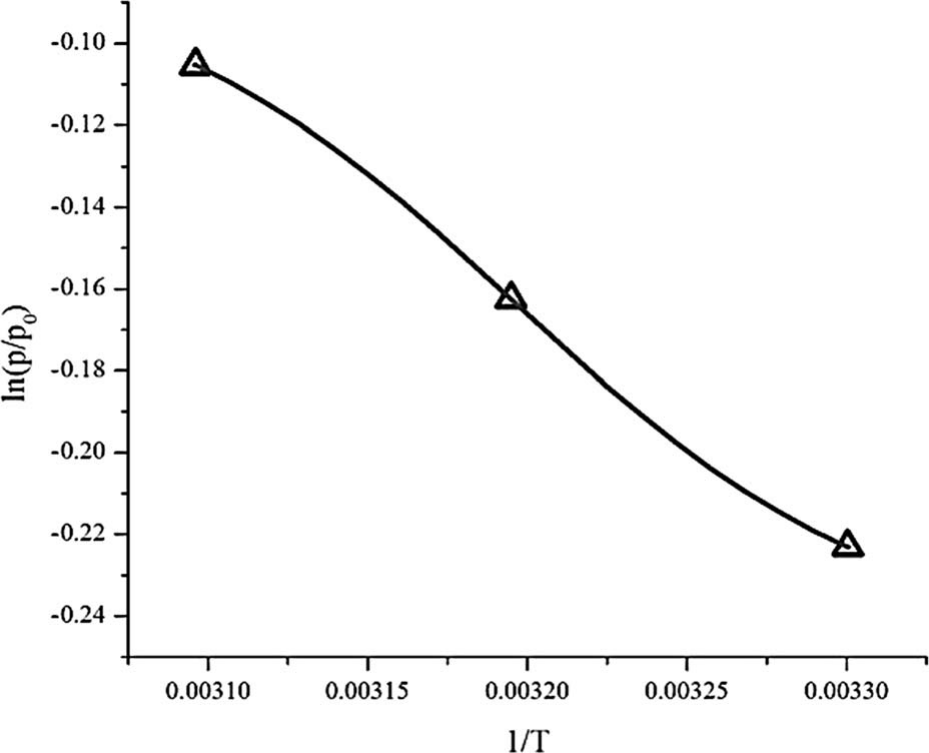
Graph of thermodynamic adsorption.

Thermodynamic parameters such as enthalpy (Δ*H*), entropy (Δ*S*) and the change in the Gibbs free energy (Δ*G*) were obtained from eqn (6). Plotting ln(*p*/*p*_0_) *versus* 1/*T* gives a slope equal to the enthalpy (Δ*H*) and an intercept equal to the entropy (Δ*S*), as can be seen in Fig. 13. The results are summarized in Table 5.

**Table:** The thermodynamic parameters of CO_2_ adsorption on zeolite-Y-templated carbon^a^

Temperature	Δ*H* (kJ mol^−1^)	Δ*S* (J mol^−1^)	Δ*G* (kJ mol^−1^)
30 °C	−4.79	−13.96	−0.56
40 °C			−0.42
50 °C			−0.28

a Δ*S* = the change in entropy. Δ*H* = the change in enthalpy. Δ*G* = the change in Gibbs free energy.

Adsorption heat (enthalpy) shows the interaction power between the adsorbate and adsorbent.^24^ From the experimental data, the enthalpy (Δ*H*) was −4792 kJ mol^−1^. The negative value of the enthalpy shows that the adsorption process was exothermic. The value of enthalpy is <80 kJ mol^−1^, which means the adsorption process of CO_2_ on the ZTC was controlled *via* physisorption.^4^ Chemical bonding between the adsorbent and adsorbate does not exist, but the interactions are due to the differences of dipole–dipole on the adsorbent surface to the atoms in the adsorbate. Generally, the enthalpy was influenced by the amount of gas that covered the adsorbent surface. When the amount of adsorbate that covered the adsorbent surface was low, there was a strong interaction between the adsorbent and adsorbate.

The entropy change (−Δ*S*) obtained was 13.959 J K^−1^ mol^−1^. The negative value of entropy suggests a decrease in randomness at the gas–solid interface during the adsorption process.^24,34^ This means that the mobility of the CO_2_ gas is limited inside the ZTC pore.

The Gibbs free energy change (Δ*G*) has a negative value, as can be seen at Table 5. This negative value indicates that the adsorption process was spontaneous without any external energy. The Gibbs free energy change values increase as the temperature of adsorption increases. This showed that at higher temperature the adsorption of CO_2_ is less spontaneous which agreed with the kinetics data.

### The structure relation of ZTC toward CO_2_ adsorption–desorption performance

**Fig. 14:**
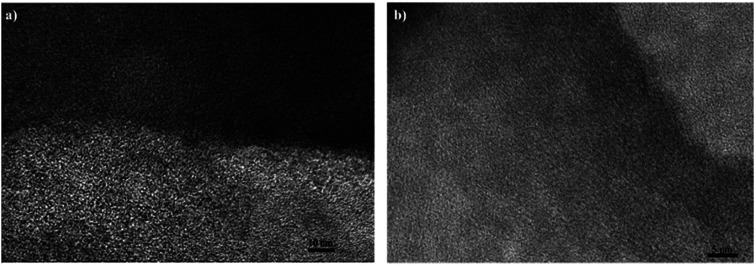
The HRTEM image of (a) the outer and inner surface of ZTC and (b) the inner surface of ZTC.

To sum up our study, a comprehensive study of the ZTC structure was conducted using HRTEM. Fig. 14a shows the outer layer of the ZTC, the brighter region in the image, and the inner surface of ZTC, the darker region in the image. The outer layer came from the unsuccessful sucrose impregnation, as discussed above. This outer layer mostly consisted of random pore structure orientation, bigger than 1 nm in size. This region was responsible for accelerating the CO_2_ transfer into the inner micropores of the ZTC due to its big pore. When moving a little bit deeper into the ZTC, the pore orientation changed into a more ordered structure. This ordered, straight, worm-like, stacked graphene structure was responsible for the majority of where the CO_2_ molecules adsorbed at a lower temperature, with a micropore size below 1 nm. Moreover, Fig. 14b shows a more detailed inner region of the ZTC. It can clearly be seen that the micropores were ordered and interconnected. This regular pore structure assisted in reaching the CO_2_ adsorption–desorption equilibrium faster and was somewhat similar to the structure of the zeolite-Y reported by Iyoki *et al.*^35^

## Conclusions

In this paper, ZTC has been synthesized as a CO_2_ capturer. The ZTC was synthesized *via* three steps: sucrose impregnation, carbonization and template removal. The results showed that the adsorption capacity was 9.51 ± 0.48 wt%, 5.60 ± 0.28 wt%, 3.47 ± 0.17 wt% or 2.39 mmol g^−1^, 1.35 mmol g^−1^, 0.82 mmol g^−1^ at temperatures of 30 °C, 40 °C, and 50 °C, respectively. The amounts of CO_2_ desorbed at temperatures of 30 °C, 40 °C, and 50 °C were up to 59.83%, 69.70%, 77.5%, respectively. Multiple runs showed that the adsorption process of CO_2_ at temperatures of 30 °C and 40 °C follows the pseudo-second order model with the highest degrees of determination (*R*^2^) of 0.972 and 0.967, while at 50 °C follows intra-particle diffusion with a degree of determination (*R*^2^) of 0.923. The thermodynamic analyses determined the change of enthalpy (Δ*H*) and the change of entropy (Δ*S*) of −4.791 kJ mol^−1^ and −13.959 J K^−1^ mol^−1^, respectively. The free energy changes at temperatures of 30 °C, 40 °C, and 50 °C were −0.562 kJ mol^−1^, 0.422 kJ mol^−1^, and −0.283 kJ mol^−1^, respectively. The adsorption process was exothermic as the value of (Δ*H*) was negative. The negative sign in the entropy change (Δ*S*) indicates the decreasing randomness on the gas–solid surface during the adsorption process. The Gibbs free energy change (Δ*G*) at temperatures of 30 °C, 40 °C, and 50 °C were −0.562 kJ mol^−1^, −0.422 kJ mol^−1^, and −0.283 kJ mol^−1^, respectively. The negative values of (Δ*G*) indicate that the adsorption process was spontaneously. With the micro–meso pore structure, this material is a good candidate for fast CO_2_ adsorption–desorption applications.

## Conflicts of interest

There are no conflicts to declare.

## Supplementary Material

RA-008-C8RA09200A-s001
