# Supplementary material for: Adsorption–desorption of CO2 on zeolite-Y-templated carbon at various temperatures
Source: RSC Adv. 2018 Dec 12;8(72):41594–602. doi: 10.1039/c8ra09200a (PMC9091942; doi:10.1039/c8ra09200a)
Supplement: RA-008-C8RA09200A-s001 [file RA-008-C8RA09200A-s001.pdf]

Electronic Supplementary Material (ESI) for RSC Advances.
This journal is © The Royal Society of Chemistry 2018

Supplementary Data

# Adsorption-Desorption of $CO_2$ on Zeolite-Y Templated Carbon at Various Temperature

Triyanda Gunawan[a], Rika Wijiyanti[a], and Nurul Widiastuti[a*]

[a] Department of Chemistry, Faculty of Science, Institut Teknologi Sepuluh Nopember, 60111 Surabaya, Indonesia

*corresponding author: nurul_widiastuti@chem.its.ac.id

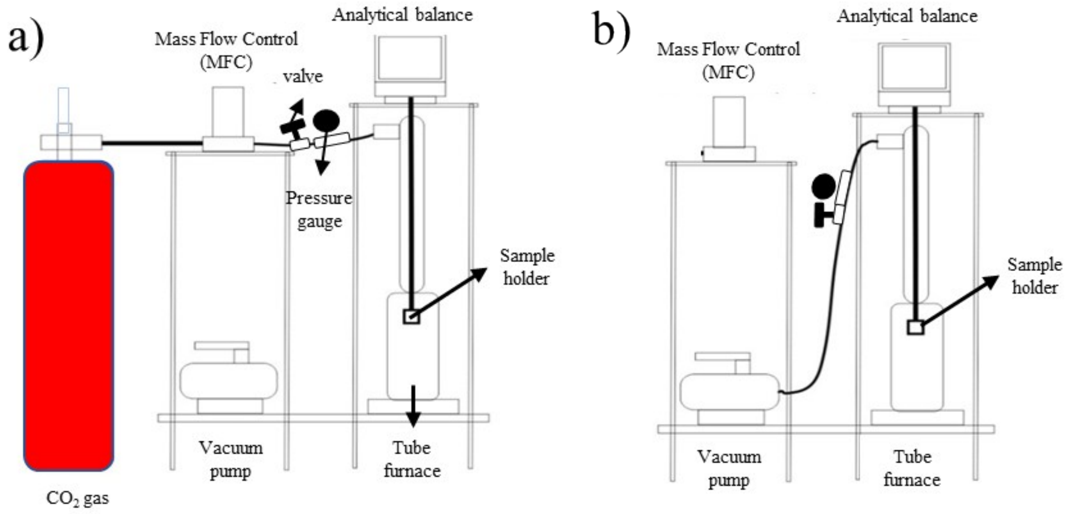


Fig S1 Schematic diagram of a) adsorption system, and b) desorption system

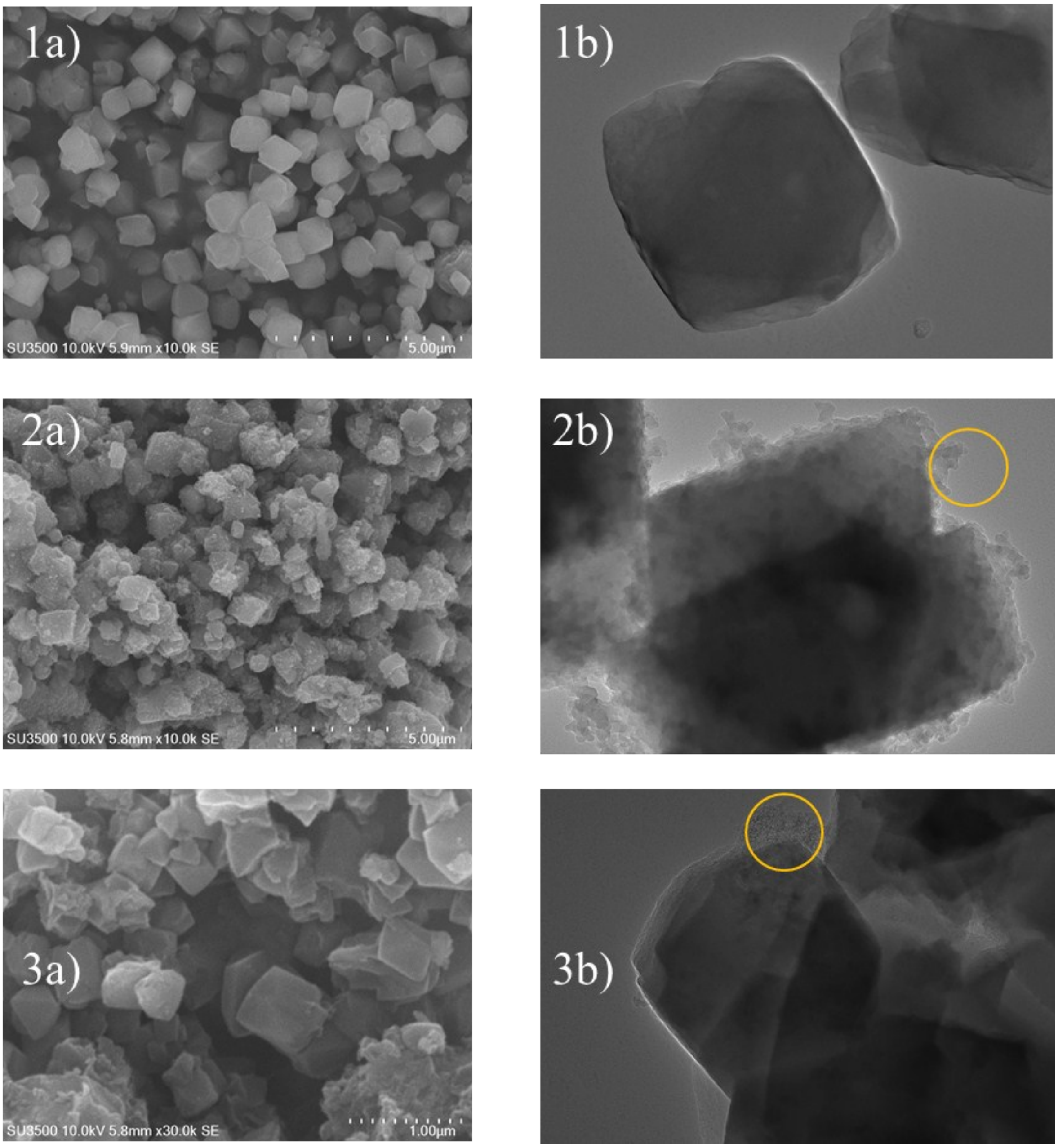


Fig S2 SEM (a) and TEM (b) image of zeolite-Y (1), composite carbon (2), and ZTC (3). Yellow circles correspond to the external graphitic carbon

The Fig S2, showing a similarity in particle shape of zeolite-Y, composite and ZTC. The octahedral structure of zeolite-Y still remained even after impregnation, carbonization and template removal.

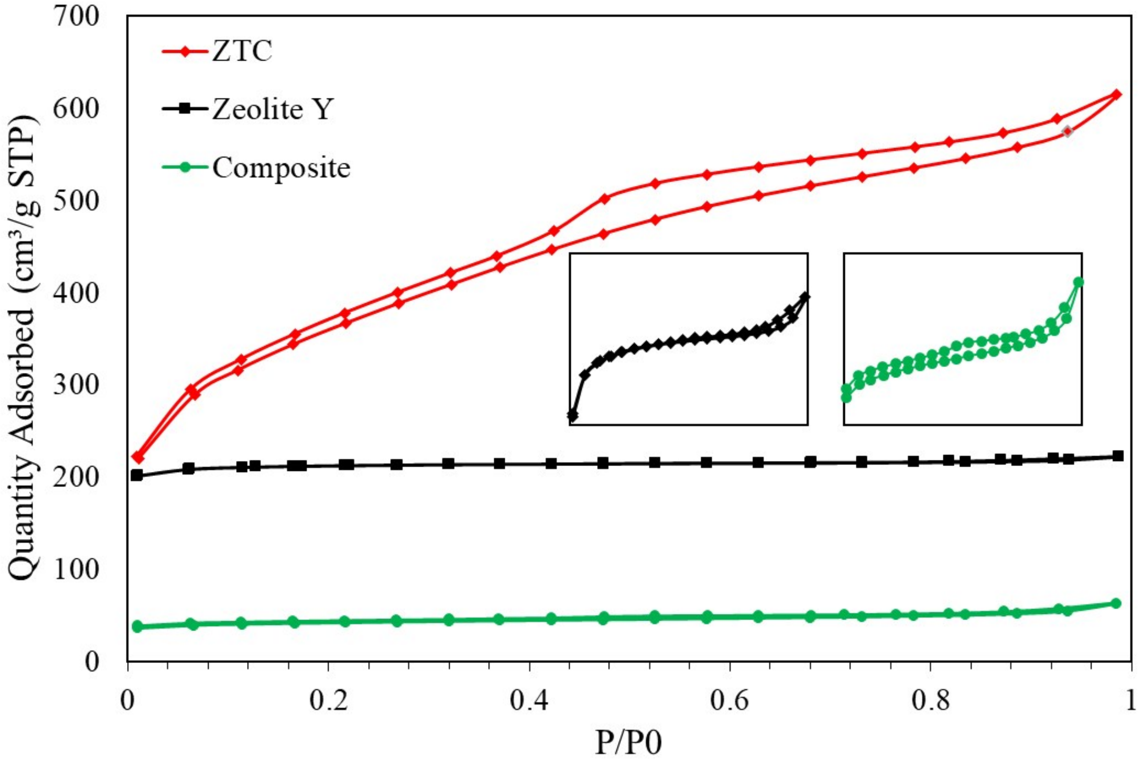


Fig S3 The $N_2$ isotherm graph of zeolite-Y, composite and ZTC

The $N_2$ isotherm showing a superior $N_2$ adsorbed on ZTC compared to the zeolite-Y and composite. The production of carbon via hard template method with zeolite was suitable method to obtain a high surface area and ordered pore structure of carbon.

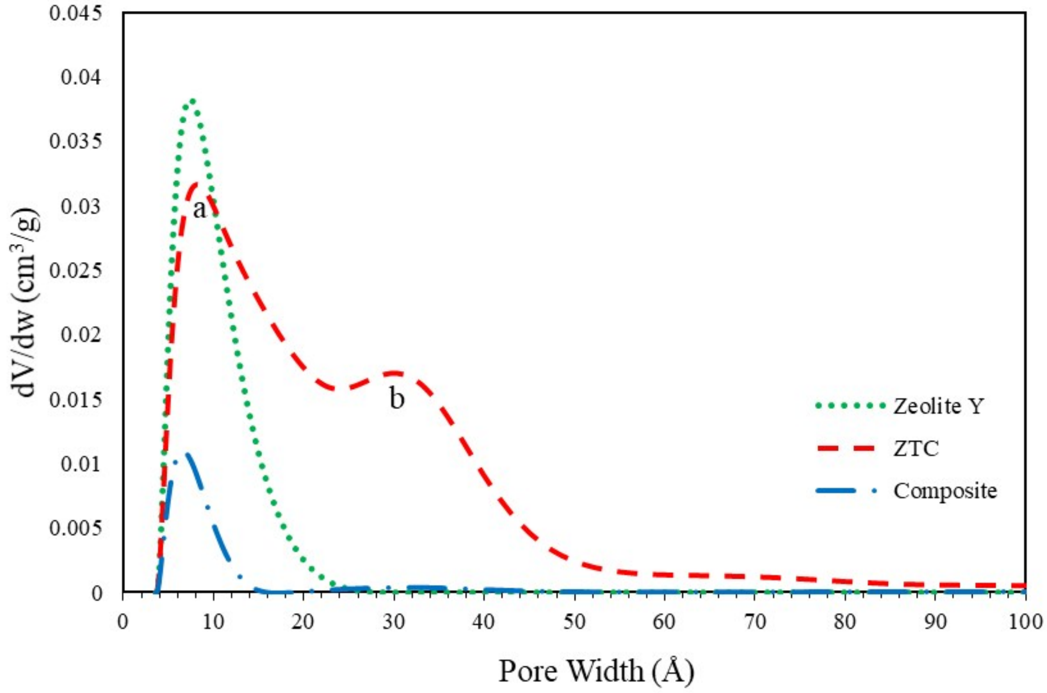


Fig S4 The PSD obtained from 2D-NLDFT calculation

The PSD obtained from SAIEUS software indicate that composite pore was filled by carbonized sucrose molecule which is noticed by the reduction average pore diameter and total $N_2$ volume adsorbed. Moreover, this method was successfully produced carbon with micro-meso structure.

**Table S1 The physical properties of all sample**

| Parameters | ZTC | Composite | Zeolite-Y |
|---|---|---|---|
| SBET ($m^2/g$) | 1254.3831 | 133.2933 | 678.48 |
| t-Plot Micropore Area | 1051.7182 | 96.0035 | 620 |
| Pore volume (cc/g) | 0.949935 | 0.097795 | 0.344 |
| Average Pore size (nm) | 1.55±0.64 | 0.729±0.04 | 0.861±0.07 |

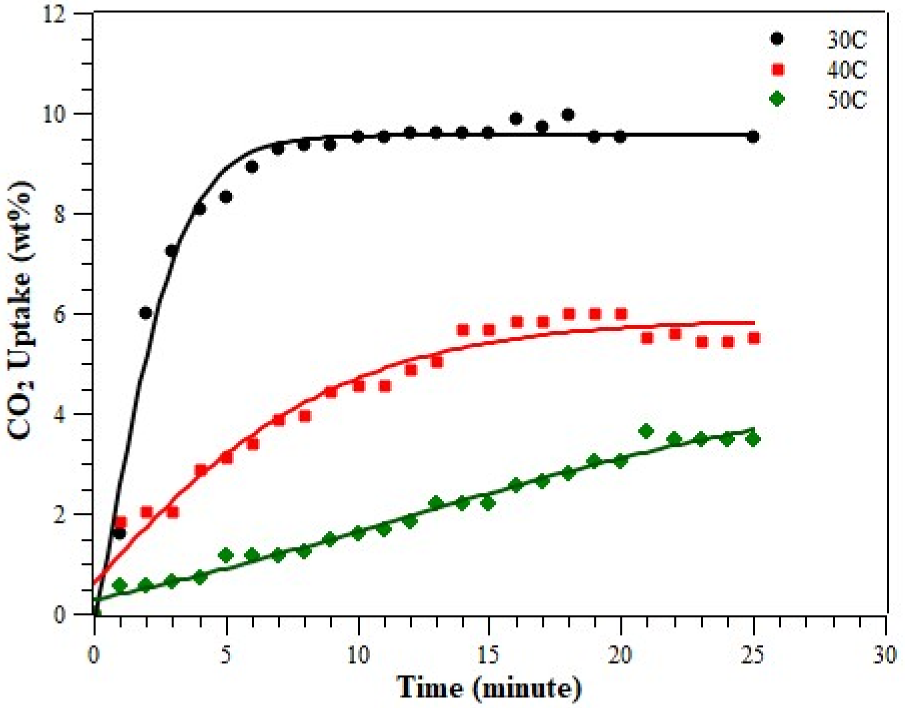


**Fig S5 The adsorption of $CO_2$ on ZTC at various temperature and a pressure of 1 Bar**

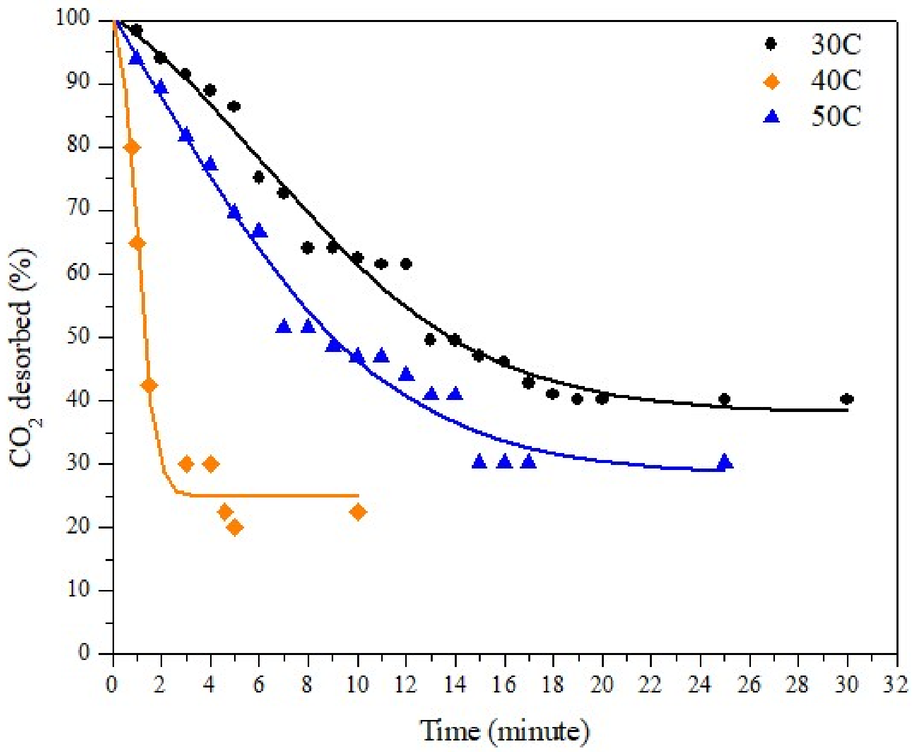


**Fig S6 Desorption of $CO_2$ on ZTC at various temperature and pressure of 1 bar**

**Table S2 Parameters of each kinetic models**

| **Model** | **Parameters** | | |
|---|---|---|---|
| Pseudo-first order | $k_f$ | $q_e$ | $R^2$ |
| 30°C | -0.036 | 1 | 0.282 |
| 40°C | -0.065 | 1 | 0.145 |
| 50°C | -0.081 | 1 | 0.763 |
| Pseudo-second order | h | $q_e$ | $R^2$ |
| 30°C | 1.663 | 2.66 | 0.972 |
| 40°C | 0.357 | 1.65 | 0.950 |
| 50°C | 0.061 | 1.47 | 0.469 |
| Intra-particle Diffusion | $k_d$ | C | $R^2$ |
| 30°C | 0.237 | 0.38 | 0.709 |
| 40°C | 0.134 | 0.10 | 0.877 |
| 50°C | 0.124 | -0.17 | 0.923 |

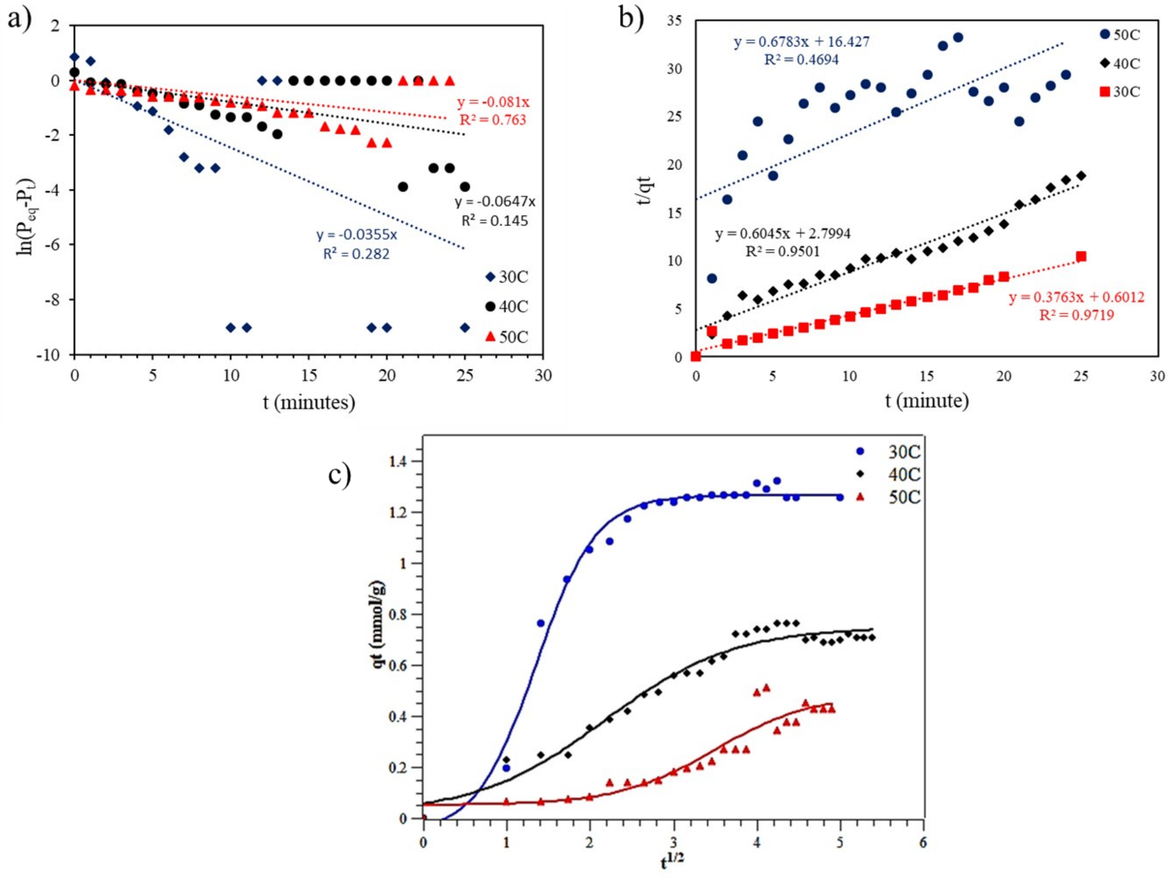


Fig S7 Graph of a) Pseudo-first order b) Pseudo-second order and c) Intra-particle diffusion

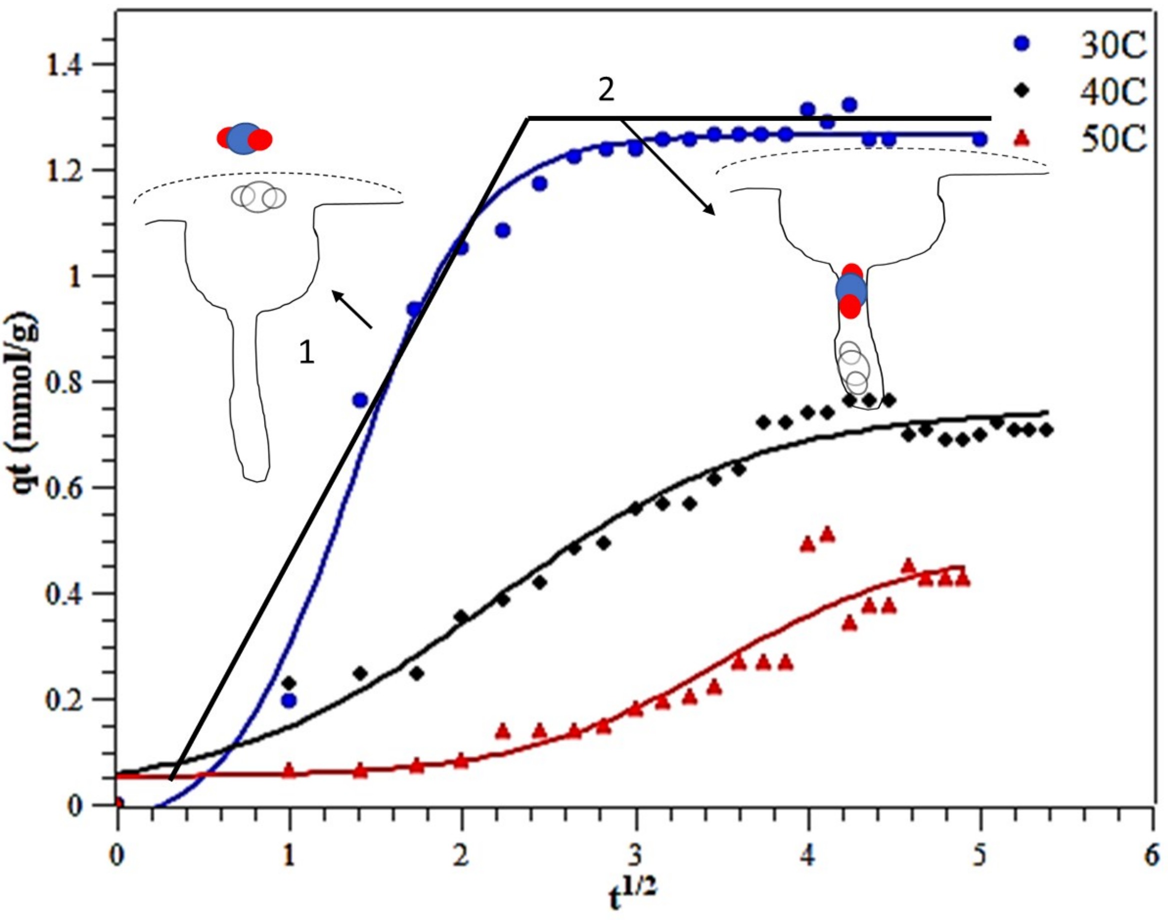


**Fig S8 Proposed $CO_2$ transfer mechanism into ZTC from intraparticle diffusion model**

The intra particle diffusion showing two steps of $CO_2$ adsorption mechanism. The first, the $CO_2$ molecule approaching the outer surface of the ZTC up to some point where the molecule movement was not limited by barrier. The second, $CO_2$ molecule making some adjustment in side the micropore and this step took longer time compared earlier steps.

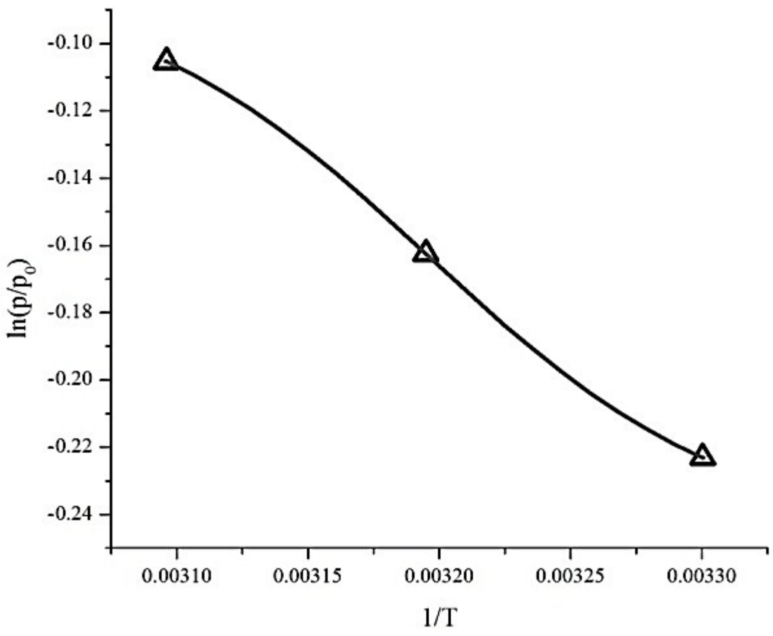


**Fig S9 Graph of thermodynamic adsorption**

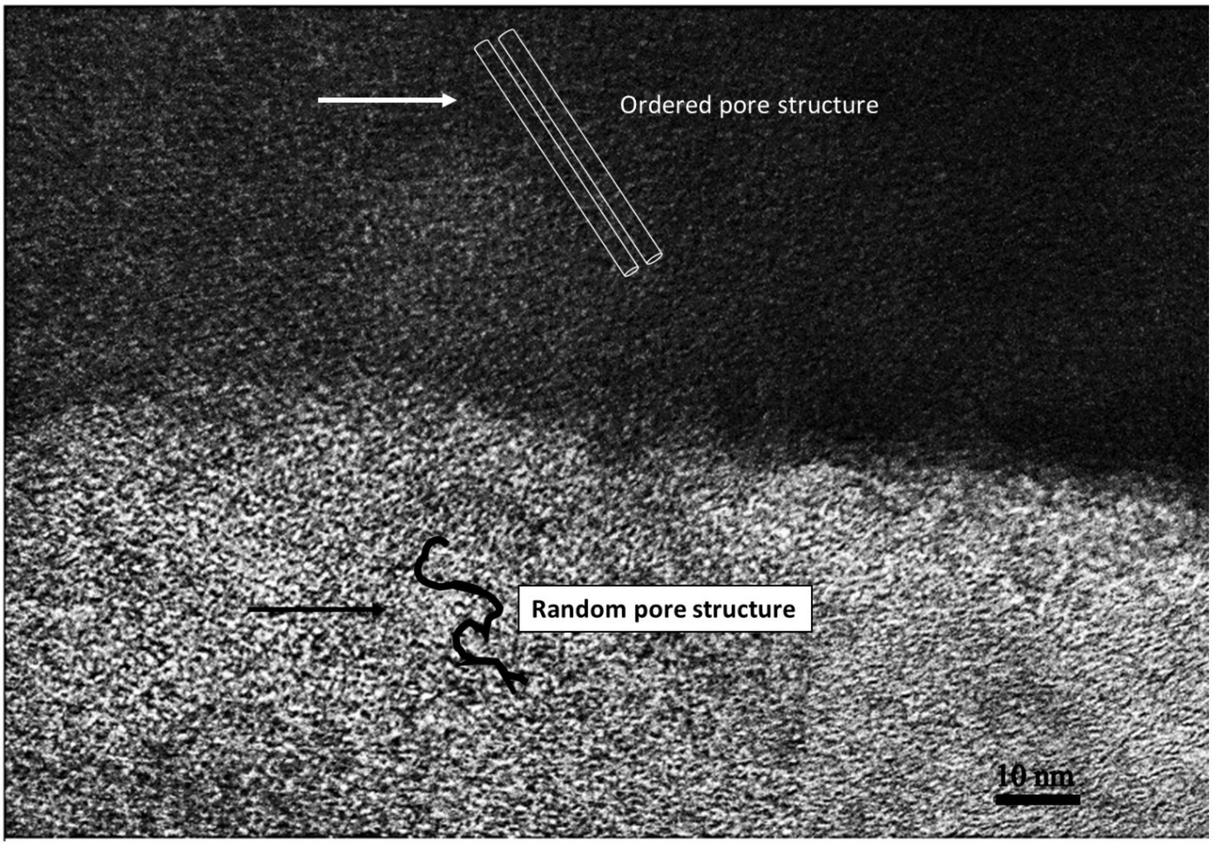


**Fig S10 High contrast HRTEM image of ZTC**

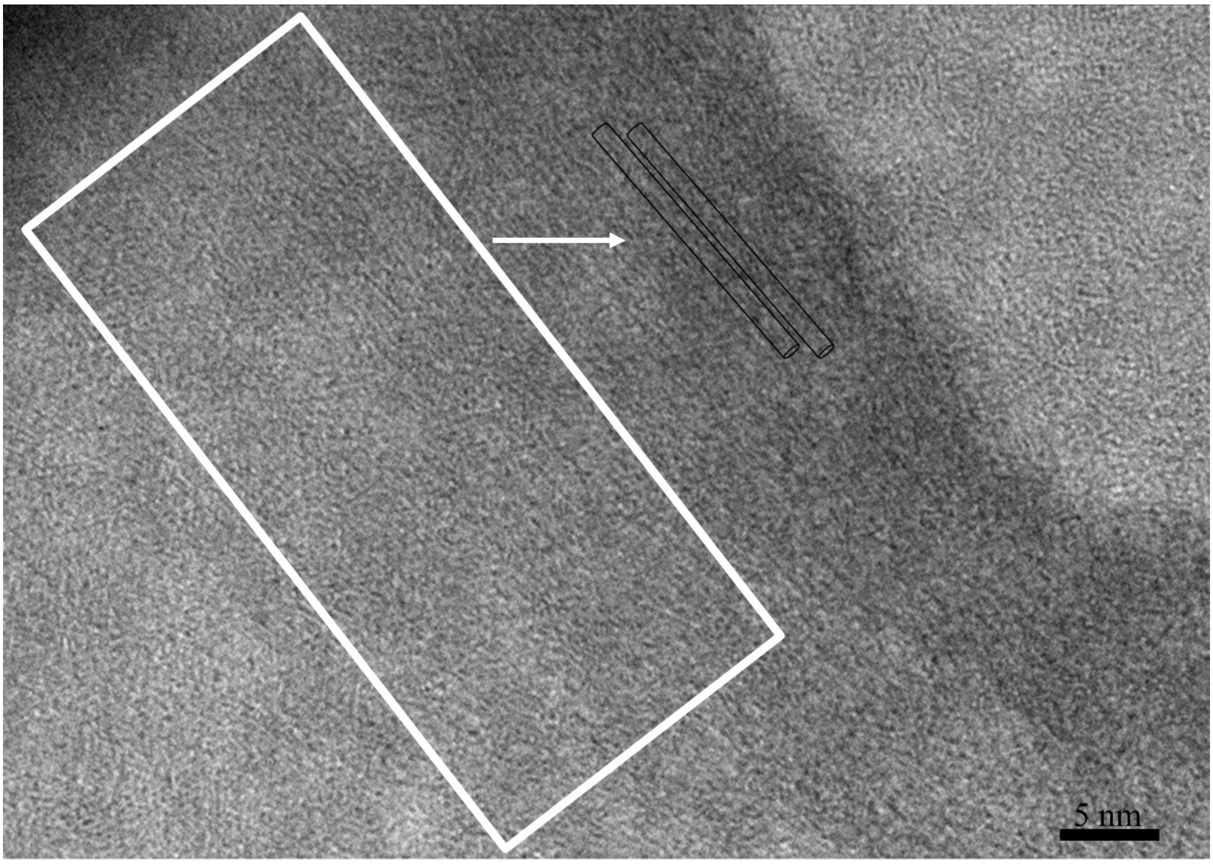


**Fig S11 The Inner pore structure of ZTC**

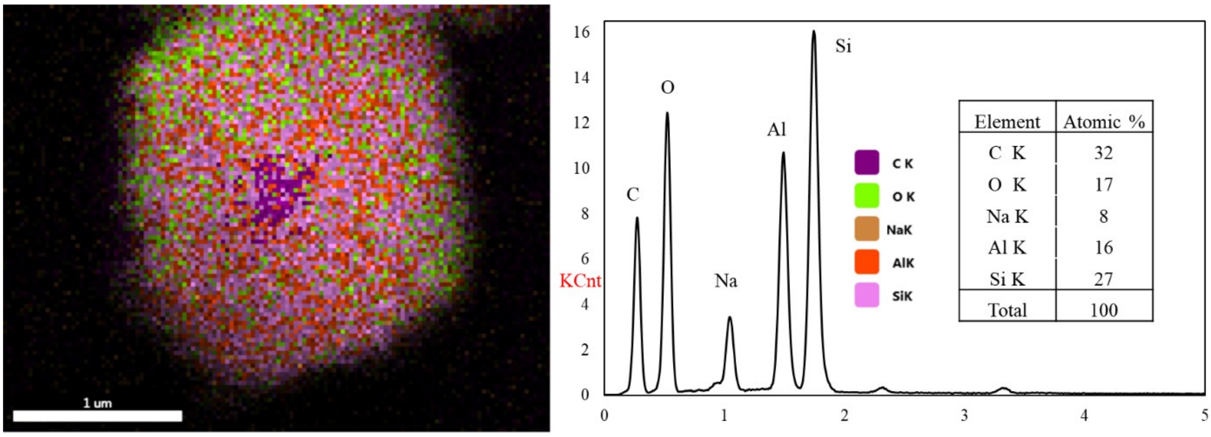


**Fig S12 The EDX mapping of composite sample**

The mapping indicated that most carbon occupied inner body of zeolite-Y

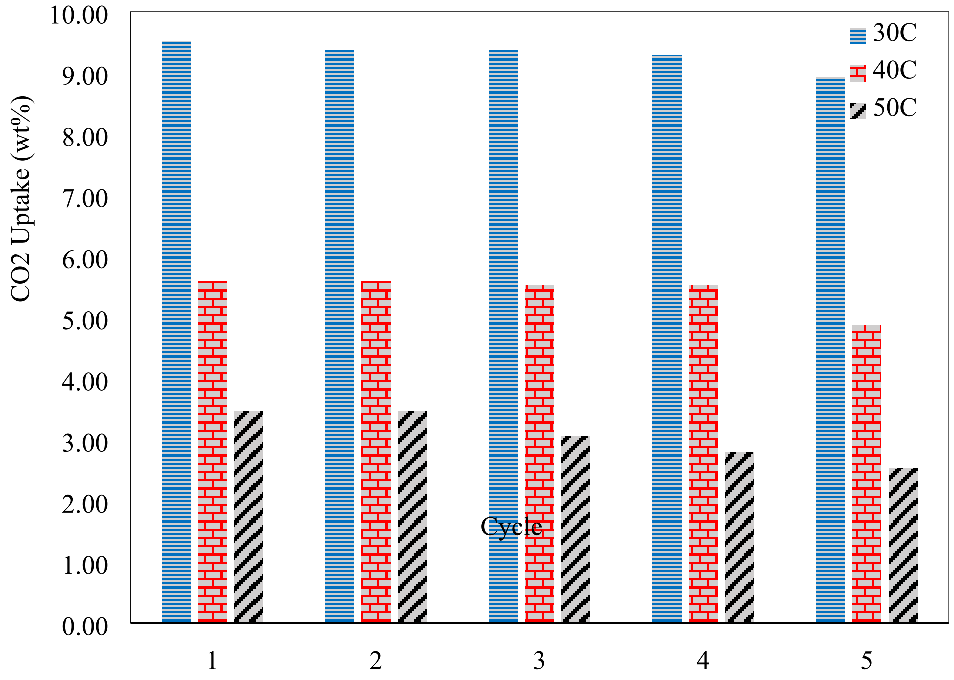


**Fig S13 The $CO_2$ uptake capacity after five cycle**

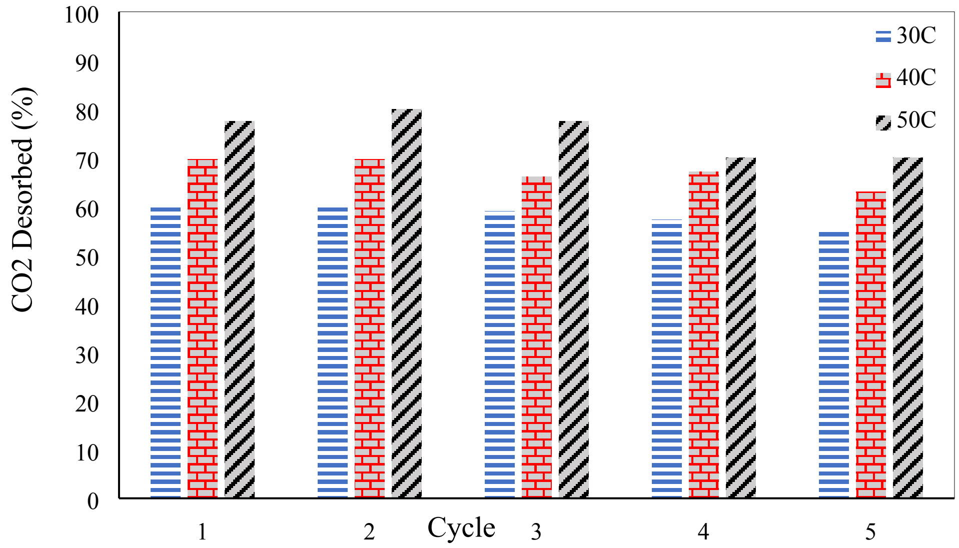


**Fig S14 The $CO_2$ desorption capacity after five cycle**
